# The Prognostic Role of Baseline Metabolic Tumor Burden and Systemic Inflammation Biomarkers in Metastatic Castration-Resistant Prostate Cancer Patients Treated with Radium-223: A Proof of Concept Study

**DOI:** 10.3390/cancers12113213

**Published:** 2020-10-31

**Authors:** Matteo Bauckneht, Sara Elena Rebuzzi, Alessio Signori, Maria Isabella Donegani, Veronica Murianni, Alberto Miceli, Roberto Borea, Stefano Raffa, Alessandra Damassi, Marta Ponzano, Fabio Catalano, Valentino Martelli, Cecilia Marini, Francesco Boccardo, Silvia Morbelli, Gianmario Sambuceti, Giuseppe Fornarini

**Affiliations:** 1 Nuclear Medicine, IRCCS Ospedale Policlinico San Martino, 16132 Genova, Italy; cecilia.marini@unige.it (C.M.); silviadaniela.morbelli@hsanmartino.it (S.M.); sambuceti@unige.it (G.S.); 2 Medical Oncology Unit 1, IRCCS Ospedale Policlinico San Martino, 16132 Genova, Italy; saraelena89@hotmail.it (S.E.R.); murianni.veronica@gmail.com (V.M.); roby.borea@gmail.com (R.B.); alessandra.damassi@gmail.com (A.D.); catalan.fab@gmail.com (F.C.); martellivalentino91@gmail.com (V.M.); giuseppe.fornarini@hsanmartino.it (G.F.); 3 Department of Health Sciences (DISSAL), University of Genova, Largo R. Benzi 10, 16132 Genova, Italy; alessio.signori.unige@gmail.com (A.S.); isabella.donegani@gmail.com (M.I.D.); albertomiceli23@gmail.com (A.M.); Stefanoraffa@live.com (S.R.); m.ponzano@campus.unimib.it (M.P.); 4 CNR Institute of Molecular Bioimaging and Physiology (IBFM), 20090 Segrate (MI), Italy; 5 Academic Unit of Medical Oncology, IRCCS Ospedale Policlinico San Martino, 16132 Genova, Italy; fboccardo@unige.it; 6 Department of Internal Medicine and Medical Specialties (DiMI), School of Medicine, University of Genova, 16132 Genova, Italy

**Keywords:** metastatic castration resistant prostate cancer, radium-223, neutrophil-to-lymphocyte ratio, lymphocyte-to-monocyte ratio, platelet-to-lymphocyte ratio, systemic inflammation index, 18F-fluorodeoxyglucose, metabolic tumor volume, total lesion glycolysis, positron emission tomography

## Abstract

**Simple Summary:**

Radium-223 is an alpha-emitting radioisotope that selectively binds to increased bone turnover areas, such as metastatic sites, acting as a bone-seeking calcium mimetic drug. Its therapeutic function in metastatic castration-resistant prostate cancer patients relies on its capability to prolong overall survival, improve quality of life, and delay the first skeletal-related event. However, in the last few years, many studies showed that the survival benefit in the real-life patients might be lower than that initially reported, probably due to a suboptimal selection of patients with poorer prognostic clinical characteristics. In this scenario, it has emerged the urgent need for the identification of reliable biomarkers able to potentially identify patients most likely to benefit from Radium-223 since baseline. With this aim, this preliminary study is the first to combine the prognostic power of baseline FDG-PET/CT and systemic inflammation indexes in a cohort of metastatic castration-resistant prostate cancer patients undergoing Radium-223 administration.

**Abstract:**

Over the last years has emerged the urgent need for the identification of reliable prognostic biomarkers able to potentially identify metastatic castration-resistant prostate cancer (mCRPC) patients most likely to benefit from Radium-223 (Ra-223) since baseline. In the present monocentric retrospective study, we analyzed the prognostic power of systemic inflammation biomarkers and 18F-Fluorodeoxyglucose Positron Emission Tomography/Computed Tomography (FDG-PET)-derived parameters and their potential interplay in this clinical setting. The following baseline laboratory parameters were collected in 59 mCRPC patients treated with Ra-223: neutrophil-to-lymphocyte ratio (NLR), derived NLR (dNLR), lymphocyte-to-monocyte ratio (LMR), platelets-to-lymphocyte ratio (PLR), and systemic inflammation index (SII), while maximum Standardized Uptake Value, Metabolic Tumor Volume (MTV), and Total Lesion Glycolysis (TLG) were calculated in the 48 of them submitted to baseline FDG-PET. At the univariate analysis, NLR, dNLR, MTV, and TLG were able to predict the overall survival (OS). However, only NLR and MTV were independent predictors of OS at the multivariate analysis. Additionally, the occurrence of both increased NLR and MTV at baseline identified mCRPC patients at higher risk for lower long-term survival after treatment with Ra-223. In conclusion, the degree of systemic inflammation, the quantification of the metabolically active tumor burden and their combination might represent potentially valuable tools for identifying mCRPC patients who are most likely to benefit from Ra-223. However, further studies are needed to reproduce these findings in larger settings.

## 1. Introduction

Bone metastases affect more than 90% of patients with metastatic castration-resistant prostate cancer (mCRPC) patients and 20–50% of them develop skeletal-related events (SREs), which represent the main cause of impaired quality of life and death [1]. 

Radium-223 (Ra-223) is an alpha-emitting radionuclide which selectively binds to areas of increased bone turnover, such as metastatic sites, acting as a bone-seeking calcium mimetic drug [2]. Its short-range high-energy emission induces breaks in double-strand DNA filaments and targets tumor cell death [3]. The phase III Alpharadin in Symptomatic Prostate Cancer Patients (ALSYMPCA) trial investigated Ra-223 compared to a placebo in mCRPC patients with symptomatic bone metastases, limited lymph node metastases (<3 cm), and no visceral metastases [4]. In the ALSYMPCA trial, Ra-223 was demonstrated to prolong overall survival (OS), improve quality of life, and delay the first SRE [4]. According to these results, Ra-223 was subsequently approved by the Food and Drug Administration (FDA) for mCRPC patients. 

However, in the last few years, many retrospective studies showed that the survival benefit in real-life patients might be lower than that reported in the ALSYMPCA trial, probably due to a suboptimal selection of patients with poorer prognostic clinical characteristics [5,6,7]. Furthermore, in 2018 the European Medicine Agency (EMA) restricted the use of Ra-223 to patients with more than six osteoblastic lesions in progression after at least two prior lines of systemic therapies for mCRPC or ineligible for any available systemic mCRPC treatment [8]. However, the later timing of Ra-223 administration in the patients’ clinical history might further negatively affect OS [9]. In this scenario, there is an urgent need to identify reliable biomarkers potentially able to improve the selection of patients most likely to benefit from Ra-223 treatment. 

Several exploratory analyses from the ALSYMPCA trial and retrospective studies identified clinical, biochemical, and imaging parameters to predict treatment completion or survival outcomes since baseline, thus potentially improving patient selection [5,6,10,11,12,13]. 

Peripheral blood inflammatory parameters, such as neutrophils-to-lymphocytes (NLR), have been shown to significantly correlate with survival outcomes and therapeutic response in various cancers, including mCRPC, as potential cancer inflammation-associated markers [14,15,16,17]. These biomarkers are currently of great interest for their ready and easy accessibility in the clinical practice and their transversal role in many types of tumors and cancer treatments [15]. However, few studies have investigated their prognostic role in patients treated with Ra-223 [5,18,19]. On the other hand, we recently showed that baseline ^18^F-Fluorodeoxyglucose Positron Emission Tomography/Computed Tomography (FDG-PET) could stratify OS in a cohort of mCRPC patients who are candidates for Ra-223 [20]. However, the comparison and the potential interplay between peripheral blood inflammatory biomarkers and metabolic FDG-PET-derived parameters still need to be investigated in this clinical setting.

Therefore, in the present proof of concept study, we analyzed the prognostic power of baseline inflammatory and functional imaging biomarkers and their potential interplay to identify mCRPC patients most likely to benefit from Ra-223.

## 2. Materials and Methods

### 2.1. Study Population

We performed a retrospective monocentric analysis of all consecutive mCRPC patients treated with Ra-223 from September 2016 to February 2020 at the IRCCS Ospedale Policlinico San Martino of Genova, Italy. The retrospective analysis was conducted under the Declaration of Helsinki, Good Clinical Practice, and local ethical and legal regulations. According to our standard procedure, all patients signed a written informed consent form, encompassing the use of anonymized data for retrospective research purposes before each imaging procedure and each Ra-223 administration. 

CRPC was defined as a serum testosterone level of <50 ng/dL following surgical or pharmaceutical castration. All patients fulfilled the inclusion criteria for Ra-223 therapy and were treated according to the standard Ra-223 regimen encompassing six intravenous administrations every four weeks (55 KBq/kg) [21]. According to the established guidelines for patient selection, a bone marrow reserve fulfilling the hematologic criteria necessary to administer Ra-223 was verified [21]. During the four weeks preceding the first Ra-223 administration, each patient underwent a contrast-enhanced CT and bone scan to select mCRPC patients with symptomatic bone metastases in the absence of visceral involvement (with the exception for lymph nodes with maximum diameter < 3 cm). In the same time interval, recruited patients were submitted to FDG-PET for prognostic purposes in agreement with the emerging prognostic role of this tool in patients with mCRPC, and in accordance with the national guidelines by the Italian Association of Medical Oncology (AIOM) [22,23]. According to our standard procedure, complete blood cell count, serum chemistry, prostate-specific antigen (PSA), alkaline phosphatase (ALP), and lactate dehydrogenase (LDH) were assessed at baseline and on the same day of each Ra-223 administration.

During Ra-223 administration, patients continued androgen deprivation therapy and received the best standard of care, including antiresorptive agents (bisphosphonates/denosumab) and antalgic therapy [21]. The concomitant treatment with Abiraterone and Enzalutamide was not allowed [21]. 

### 2.2. Systemic Inflammation Indexes 

We retrospectively collected white blood cells (WBC), platelets (PLT), and the absolute neutrophil (ANC), lymphocyte (ALC), and monocyte (AMC) count to obtain their ratio: NLR, derived NLR (dNLR), lymphocyte-to-monocyte ratio (LMR), platelets-to-lymphocyte ratio (PLR), and systemic inflammation index (SII). dNLR was calculated as ANC/(WBC-ANC) and SII as NLRxPLT. 

### 2.3. Imaging Procedures and Images Analyses

FDG-PET was performed according to the European Association of Nuclear Medicine (EANM) Guidelines [24]. PET/CT scans were performed using a 16-slices PET/CT hybrid system (Hirez-Biograph 16, Siemens Medical Solutions, USA). 

FDG-PET images were interpreted in consensus by three expert nuclear medicine physicians (M.B.; M.I.D.; A.M.) blinded to contrast-enhanced CT and bone scan results. From the attenuation corrected FDG-PET images, the maximum standardized uptake value (SUVmax) of the hottest bone lesion was obtained in the transaxial view. Further, a volume of interest was drawn using an SUV-based automated contouring program (Syngo Siemens workstation, Siemens Medical Solutions, USA) with an isocounter threshold based on 40% of the SUVmax, as previously recommended [25]. Total Metabolic Tumor Volume (MTV) was obtained by the sum of all skeletal and extra-skeletal lesions. Total Lesion Glycolysis (TLG) was calculated as the sum of the product of MTV of each lesion, and the SUVmean value, which, in turn, was automatically calculated within each single MTV. 

Aiming to analyze the interobserver variation, a second expert PET reader (S.M.) measured MTV and TLG independently from the first group of observers. 

### 2.4. Survival Assessment 

Baseline inflammatory biomarkers (NLR, dNLR, LMR, PLR, SII), as well as FDG-PET-derived parameters (SUVmax, MTV, TLG), were assessed for their correlation with OS.

### 2.5. Statistical Analysis

Descriptive analyses were conducted using percentages for binary variables and means/medians for continuous variables, reporting their dispersion values. 

Non-parametric Bland–Altman plots were used to evaluate median bias ad limits of agreement (2.5% and 97.5% percentiles) for MTV and TLG values measured by the two groups of PET readers [26]. A linear regression was applied to verify the eventual occurrence of proportional biases.

To assess the association of parameters, biomarkers, and clinical characteristics (independent variables) with OS, univariable and multivariable Cox regression model were used. 

All parameters, biomarkers, and clinical characteristics, with a *p*-value < 0.10 at univariable analysis were selected for the multivariable analysis. Only those with a *p*-value < 0.10 were maintained in the final multivariable model.

OS was calculated from the start of treatment to death from any cause, censored at last follow-up for patients who were alive, and was estimated by mean of the Kaplan–Meier (KM) approach. 

The multivariable model was performed both on the cohort with complete cases for inflammatory and FDG-PET parameters and for the whole cohort of patients after multiple imputations of missing values for FDG-PET parameters (MTV, TLG). Multiple imputation was performed using an iterative multivariate method based on chained equations. Eleven imputations were performed.

To report KM curves of OS, continuous parameters were binarized. To find the best cut-off value, the Youden index from the ROC curve for survival data at 24 months was used. 

Hazard-ratios (HR) for Cox regression models were reported together with a 95% confidence interval (CI) and *p*-value. Due to a highly skewed distribution, for MTV and TLG, the log-transformed values were used in the analyses for a better interpretation. Gleason Score at diagnosis was categorized into three classes for clinical interpretation, as previously described [27]. Similarly, bone scan lesions were categorized in <6, 6–20, and >20, as performed in a sub-analysis of the ALSYMPCA study [4].

To assess the ability of each continuous parameter to discriminate between dead and censored patients, the Harrell’s c-index for censored data was calculated for all inflammatory and FDG-PET parameters. 

Further, a calibration plot to assess the accuracy of the prediction for an individual patient according to single parameters and multivariate model was realized. A joint test was performed to investigate the overall evidence for linear miscalibration. 

All statistical analyses were performed using Stata (v.16; StataCorp; 4905 Lakeway Drive College Station, Texas, TX, USA).

## 3. Results

### 3.1. Patients’ and Treatment Characteristics

Fifty-nine mCRPC patients treated with Ra-223 were included in the analysis. Of these, 48 (81.4%) had all complete data on both inflammatory biomarkers and FDG-PET parameters. The patient, tumor, and treatment characteristics are summarized in Table 1.

All patients had a histological diagnosis of prostate cancer with a median Gleason score (GS) of 8 (range 5–10) with a GS ≥ 8 in 51% of patients; 47% of patients had metastatic disease at diagnosis.

Before Ra-223 therapy initiation, the median age was 74 years (range 51–88 years) and median ECOG PS was 1 (range 0–2), with ECOG PS 0–1 in 76% of patients. 

All patients underwent CT and bone scans at baseline, while 81% of the enrolled patient underwent FDG-PET at the same timepoint. CT scan revealed the occurrence of lymph node metastases in 27% of patients, while bone scan showed the presence of <6, 6–20, and >20 bone lesions in 16%, 40%, and 44% of patients, respectively. Baseline peripheral blood results were available in 100% of patients. 

Ra-223 therapy was administered as first and second line therapy for CRPC in 24 patients (41%), as third line in 21 (36%) patients, and as subsequent lines in 14 patients (23%). In 26 patients (44%), Ra-223 was administered after the 2018 EMA restriction of use [8]. 

The median number of Ra-223 cycles was 5 (range 1–6), 78% and 39% of patients completed three and all six cycles, respectively. Eight patients (13%) are currently still in treatment with Ra-223 at the time of data analysis. Thirty-five patients (59%) received chemotherapy before Ra-223, of which 19 patients (32%) received docetaxel only and 16 patients (27%) both docetaxel and cabazitaxel.

Characteristics of patients with complete cases were similar to those of the whole cohort.

### 3.2. Interobserver Agreement between PET Readers

There was good agreement between the two groups of observers for measuring MTV and TLG. Bland–Altman plots showed a median difference of 0.26 (limits of agreements: −159.67–160.19) and −15.35 (limits of agreements: −397.07–366.37), respectively (see also Figure S1). No proportional biases were observed (p = ns for both).

### 3.3. Systemic Inflammation Indexes and FDG-Derived Parameters in the Prediction of OS.

**Figure 1:**
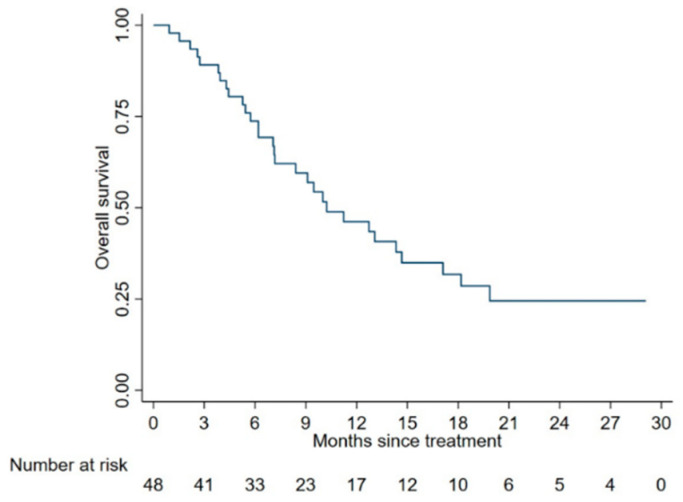
Survival function of the entire study cohort.

**Figure 2:**
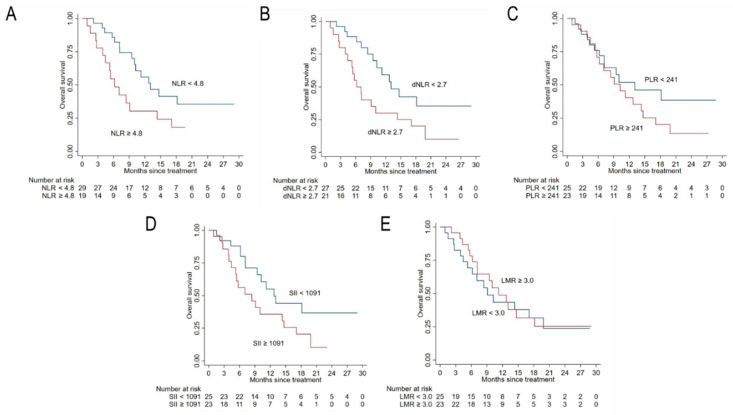
Kaplan–Meier curves for overall survival (OS) according to baseline systemic inflammatory indexes. Panels (**A**−**E**) show OS prediction according to neutrophil-to-lymphocyte ratio (NLR), derived-NLR (d-NLR), platelets-to-lymphocyte ratio (PLR), systemic inflammation index (SII), and lymphocyte-to-monocyte ration (LMR), respectively.

**Figure 3:**
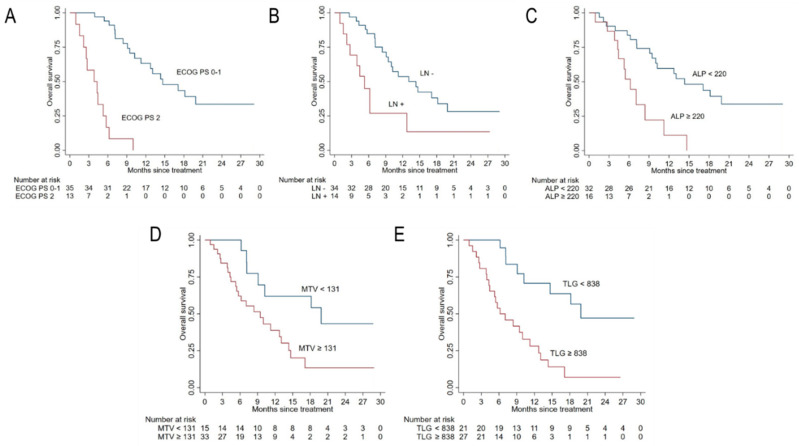
Kaplan–Meier curves for OS according to baseline clinical and FDG-PET parameters. Panels (**A**−**E**) show the prediction of OS according to ECOG PS, the presence of pathological lymph nodes (LN+), baseline ALP, MTV, and TLG, respectively.

All patients included in the study were assessable for survival analysis and were followed-up for a median of 10 months. The median OS (mOS) was 11.6 months (95% CI: 9.1–14.7) in the whole cohort and 10.2 (95% CI: 7.1–14.7) in 48 patients with complete data. OS was 78.8% (CI: 65.6–87.3) in the whole cohort and 73.4% (58.4–84.2) in the reduced sample at 6 months, while it was 46.5% (CI: 32.4–59.4) in the whole cohort and 46.2% (CI: 30.6–60.5) in the reduced sample, respectively. Figure 1 shows the Kaplan–Meier survival function of the study cohort with all complete data (*n* = 48). Results from Kaplan–Meier analyses and univariable Cox regression analyses are reported in Figure 2 and Figure 3 and in Table 2, respectively. Univariate and multivariate analyses were conducted on the complete cases set (*n* = 48). A further multivariate analysis was performed considering all sample (*n* = 59) after multiple imputation of missing data for FDG-PET parameters.

Lower ECOG PS and ALP, as well as the absence of pathological lymph nodes, were associated with higher OS. Among systemic inflammation indexes, only NLR and dNLR reached the statistical significance at the univariate analysis (Table 2, Figure 2). In both cases, higher OS was observed for lower values of these systemic inflammation parameters. Similarly, lower MTV and TLG correlated with an increased OS (Table 2, Figure 3). 

**Figure 4:**
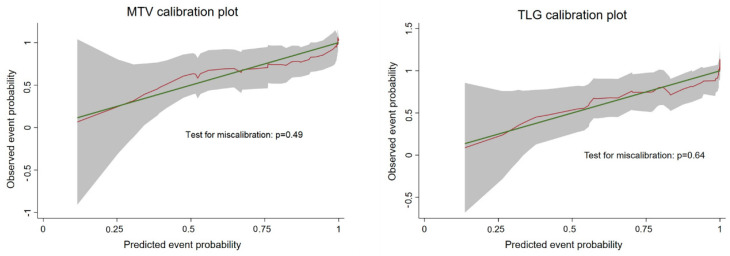
Calibration plot showing the relationship between the predicted and observed probability of death at 24 months for the two Fluorodeoxyglucose (FDG)-derived parameters. A well-calibrated parameter shows a 1:1 relationship between predicted and observed probability represented by a 45° line (green line). The predicted probability (red line) is based, respectively, on MTV (**left panel**) and TLG (**right panel**) and is remarkably close to the green line representing a good calibration. This is also confirmed by a not significant test for miscalibration.

Low to moderate *c*-index, testing the discriminative ability, was observed for almost all inflammation parameters while MTV and TLG showed a higher *c*-index of 0.75. Both these parameters also seemed to have good accuracy in prediction (Figure 4) as also confirmed by a not significant result of the test for miscalibration (*p*-value = 0.49 for MTV and 0.64 for TLG).

The same parameters with prognostic value at the univariate analysis (apart from ALP, dNLR, and TLG) remained independently associated at the multivariate analysis for OS. The Harrell’s *C*-index for this multivariable model was 0.81 in the reduced cohort (without imputation of missing value) and 0.79 in the whole cohort where FDG-PET parameters were imputed for the missing 11 patients.

### 3.4. The Combination of Systemic Inflammation Indexes and FDG–Derived Parameters in the Prediction of OS

Baseline NLR and MTV, both independently associated with OS in the Cox proportional hazard analyses, were thus combined. This allowed us to define an immune-metabolic-prognostic index (IMPI), as previously described by Castello et al. [28]. The combination of the above-mentioned parameters allowed us to identify three groups with different risk as it follows: low risk (neither NLR ≥ 4.8 nor MTV ≥ 131, IMPI = 0), intermediate risk (NLR ≥ 4.8 or MTV ≥ 131, IMPI = 1), and high risk (NLR ≥ 4.8 and MTV ≥ 131, IMPI = 2).

**Figure 5:**
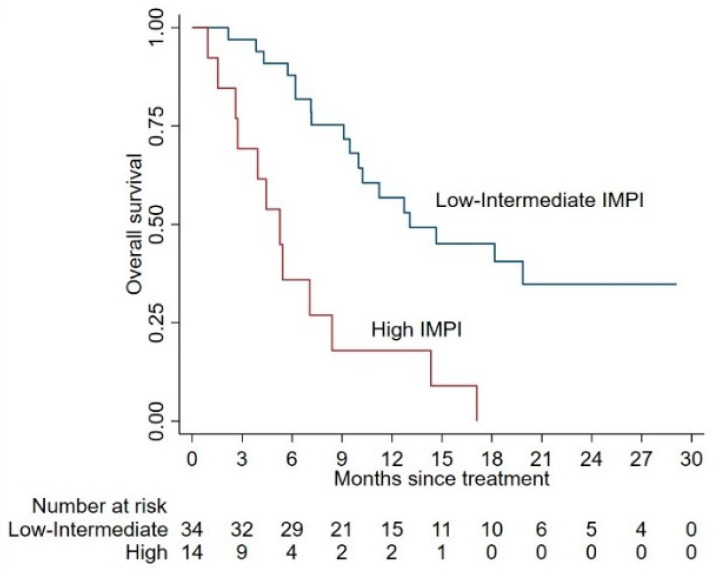
Kaplan–Meier curves for OS according to the immune-metabolic-prognostic index (IMPI). The figure shows the prediction of OS according to the IMPI, based on baseline NLR and MTV, categorizing patients in two groups: high risk (2 factors), and low-intermediate risk (0–1 factor).

Among the 48 patients evaluable for IMPI, 10 (20.8%) had low risk, 24 (50%) had intermediate risk, and 14 (29.2%) had high risk. Median OS was 18.2 months (95% CI 7.1–30 months), 12.7 months (95% CI 9.1–25.3 months), 5.3 months (95% CI 2.6–8.4 months) for the low, intermediate, and high IMPI groups, respectively. While no significant differences were observed between low and intermediate groups (*p* = 0.27), the high IMPI group was significantly different with respect to the remaining classes (*p* = 0.001 vs. low, and *p* = 0.001 vs. intermediate). Results from the Kaplan–Meier analysis of IMPI are reported in Figure 5. The prognostic power of IMPI was confirmed including this score in a multivariable model incorporating ECOG-PS, and lymph node metastases (*p* = 0.001). 

## 4. Discussion

Ra–223 is one of the therapeutic options for mCRPC patients and was approved after the survival benefit observed in the phase III ALSYMPCA trial (mOS 15 versus 11 months) [4]. Nonetheless, in real-life experience, lower survival outcomes (mOS ranging from 8 to 13 months) compared to the results of the registration trial were observed [5]. This weaker survival benefit might be at least partially related to the suboptimal patients’ selection process, which was further complicated by the restriction of the use of Ra-223 started in 2018 [29]. In fact, mCRPC candidates to Ra-223 therapy have weaker clinical characteristics compared to those included in clinical trials. Moreover, among the treatment options for mCRPC patients, the right collocation of Ra-223 treatment is not well-established as no comparative and sequential clinical trials are currently available [19]. There is, therefore, an unmet need to identify baseline clinical, biochemical, or imaging biomarkers able to improve the prognostic stratification of patients undergoing Ra-223. 

In recent years, many studies tried to identify biomarkers to better select patients most likely to benefit from Ra-223 and, therefore, to optimize treatment strategies. These are important to gain as much as possible in efficacy with few side effects, to improve survival outcomes of mCRPC and healthcare costs. Widely studied clinical variables with prognostic value in Ra-223 patients included ECOG PS, previous lines of therapy and prior chemotherapy [6,7,12,30]. Furthermore, the number of Ra-223 administered cycles was associated with OS [30,31]. Among laboratory variables, baseline PSA, LDH, and, especially, ALP (as an indirect index of disease burden) and hemoglobin (as an index of bone marrow reserve) have been shown to provide relevant prognostic insights in these patients [6,7,11,12,30,32]. 

Similar to previous studies, in our patient’s cohort we observed a lower mOS compared to that reported in the ALSYMPCA trial [4]. Moreover, the independent prognostic value of baseline clinical variables such as the ECOG PS, the presence of lymph node metastases and ALP levels was confirmed. Unlike previous studies, we extended our analysis to systemic inflammatory biomarkers as well as to FDG-PET-derived parameters.

Tumor microenvironment and systemic inflammation are known to influence therapeutic response and clinical outcomes [33,34]. Hence, many inflammatory biomarkers are currently under investigation as tools to predict the therapeutic effect or prognosis in different types of advanced cancer [35]. NLR is the most studied, and it is widely established that higher levels of NLR predict poor OS regardless of the tumor type, stage and treatment [14,36]. Other types of inflammatory biomarkers were also assessed for their prognostic role in cancer [37,38,39,40]. Among them, NLR, PLR and SII have been shown to be prognostic in mCRCP patients treated with both chemotherapy or new-generation hormonal agents [16,17,41,42,43,44,45]. However, few data on peripheral blood biomarkers are available in mCRPC patients treated with Ra-223. In the last years, few real-world studies showed a reliable, independent disease-related prognostic power of NLR in this setting, mainly related to the prediction of OS and, to a lesser extent, of PFS [5,19,46]. To the best of our knowledge, the present study represents the first analysis of different types of inflammatory biomarkers as prognostic factors in mCRPC patients undergoing Ra-223. However, while both NLR and dNRL were characterized by a prognostic power at the univariate analysis, only a lower baseline NLR was independently associated with longer OS. 

On the other hand, FDG-PET-derived parameters displaying the extent (MTV) and the intensity (TLG) of the metabolically active disease burden were predictors of OS. This result reproduces our previous study, which, however, was conducted on a smaller patient sample [20]. Furthermore, in the present study, the multivariate Cox regression analysis showed that the prognostic power of MTV was independent of the one provided by the degree of systemic inflammation.

**Figure 6:**
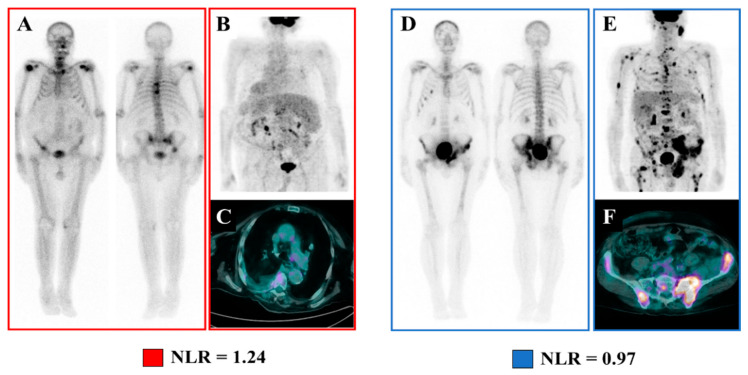
An emblematic example of a mismatch between the metabolic burden of metastatic disease and the systemic inflammation state. At baseline, these two patients showed similar degrees of metastatic burden at bone scan (Panels (**A**–**D**)) and similar NLR values, while FDG–PET showed a relevant mismatch in the extent of the metabolically active metastatic burden (Panels (**B**–**E**) show the Maximum intensity projection PET images, while Panels (**C**–**F**) show the axial section of the hottest bone lesion; MTV = 79.1 cm^3^ and 985.4 cm^3^, respectively).

On the pathophysiological ground, obtained results might improve the comprehension of the still poorly defined molecular mechanisms underlying FDG accumulation in advanced CRPC patients. Indeed, while FDG-PET is not useful in naïve prostate cancer as it shows low FDG-avidity, CRPC patients are characterized by higher levels of FDG uptake, particularly in chemotherapy-refractory patients [47]. The progressive increased FDG avidity in the later stages of CRPC might be explained (at least theoretically) by two different mechanisms. On one side, the overexpression of GLUT-1 in cell membranes and the enhanced Warburg effect characterizing PC cells in advanced stages might justify this phenomenon [48,49]. On the other hand, emerging data support the role of local inflammation and, in particular, of FDG-avid macrophage and lymphocyte recruitment in the tumor microenvironment, as tumor-promoting factors driving PC from the hormone-sensitive stage to refractivity [50,51]. The observed independence between the prognostic power of FDG-PET imaging and systemic inflammation indexes (which roughly measure the degree of tumor inflammation) might imply the prevalence of the former rather than the latter pathway as the underlying mechanism of FDG uptake in mCRPC. In this scenario, the detection of FDG-avid de-differentiated (eventually low osteoblastic) metastatic disease might mirror greater tumor aggressiveness, possibly predicting the lower Ra-223 accumulation and the consequent low-response rate, regardless of the systemic inflammation state (Figure 6) [20,52]. The same considerations also allow us to interpret the observed capability of the composite of pretreatment NLR and MTV (termed IMPI) to stratify the OS. Indeed, patients at the higher IMPI risk class might be characterized by the occurrence of both tumor microenvironment inflammation and de-differentiation, leading to a worst long-term survival and Ra-223 treatment outcome. 

Our study has several limitations. First, a major limitation is represented by its retrospective and monocentric nature and the consequent low number of patients analyzed. Indeed, the observed prognostic inadequacy of the systemic inflammatory indexes other than NLR (and its derived counterpart) might be related to the present study’s low statistical power. On the other hand, in a subgroup of 11 patients, baseline FDG-PET was not performed. We tried to overcome this limitation through the imputation of missing values. However, the current data should be considered preliminary, while a better-defined comparison between these biomarkers needs to be assessed in a larger multicentric setting. According to this, we are currently planning a retrospective multicenter study to evaluate further the prognostic and predictive value of peripheral blood biomarkers and FDG-PET in Ra-223-treated patients. In this setting, also the immune-metabolic-prognostic index will require further validation. However, despite the sample of our patients was small, it was highly representative of the population enrolled in the ALSYMCA trial [4] as it can be observed comparing the clinical characteristics of the two studies. Similarly, the prognostic role of well-known prognostic factors (ECOG, ALP, lymph node metastases) was confirmed anyway, as further proof of its representativeness. Lastly, the monocentric nature of the analyses may also represent one of the strengths of this study. In fact, all the enrolled patients were submitted to FDG imaging using the same PET/CT scanner, avoiding the possible influence of the inter-scanner variability on PET results, possibly hampering SUVmax, MTV, and TLG reproducibility [53]. Besides the dimension of the patient’s cohort, the few clinical collected variables might also represent a potential limitation of the present study. According to this, due to the intrinsic limitations of retrospective data collection, the steroid use by each patient before Ra-223 administration was not recorded and considered as a possible confounding factor. Indeed, corticosteroids might have increased baseline NLR in some patients, introducing potential bias in the data interpretation. However, Lorente et al. previously reported an independent association between baseline NLR and OS in a cohort of mCRPC patients undergoing second-line chemotherapy, regardless of the corticosteroid use at baseline [54]. Moreover, the use of corticosteroids for palliation of symptoms in advanced mCRPC with bone-involvement is well established in clinical practice, and this characteristic of the study cohort highly reflects the setting of the real-world. Larger multicentric studies are required to disclose the robustness of NLR concerning steroid administration in mCRPC patients undergoing Ra-223.

## 5. Conclusions

The degree of systemic inflammation and the quantification of the metabolically active tumor burden through FDG-PET imaging provide independent prognostic insights in mCRPC patients undergoing Ra-223. The combination of these biomarkers might represent a potentially valuable tool for identifying mCRPC patients who are most likely to benefit from Ra-223 since baseline. Larger studies are needed to further evaluate this hypothesis and, eventually, to confirm these preliminary results. 

## Figures and Tables

**Table 1:** Patients’ characteristics.

Patients’ Characteristics	All Sample	Complete Cases
	(*n* = 59)	(*n* = 48)
Clinical characteristics	*n (%)*	*n (%)*
Median age, years (range)	74 (51–88)	75 (51–88)
ECOG performance status		
0	22 (37)	17 (35)
1	23 (39)	18 (38)
2	14 (24)	13 (27)
Gleason score at diagnosis		
≤7	21 (36)	16 (33)
≥8	30 (51)	24 (50)
Missing data	8 (13)	8 (17)
Gleason group at diagnosis		
≤2	10 (17)	8 (17)
≥3	42 (71)	33 (69)
Missing data	7 (12)	7 (14)
Prostatectomy		
Yes	21 (36)	17 (35)
No	35 (59)	28 (58)
Missing data	3 (5)	3 (7)
Radical radiotherapy		
Yes	4 (7)	3 (6)
No	48 (81)	40 (83)
Missing data	7 (12)	5 (11)
Metastatic disease at diagnosis		
Yes	28 (47)	22 (46)
No	27 (46)	23 (48)
Missing data	4 (7)	3 (6)
Metastases		
Bone metastases	43 (73)	35 (73)
Bone and lymph node metastases	16 (27)	13 (27)
N bone metastases		
<6	9 (16)	8 (16)
6–20	24 (40)	20 (42)
>20	26 (44)	20 (42)
Baseline median PSA, g/L (range)	55 (0–6089)	68 (0–6089)
Baseline median ALP, U/L (range)	138 (10–1296)	154 (29–1296)
Baseline ALP, U/L		
<220	40 (68)	32 (67)
≥220	19 (32)	16 (33)
Ra-223 treatment		
Ra-223 treatment line		
Median (range)	3 (1–6)	3 (1–6)
First-line	3 (5)	2 (4)
Second-line	21 (36)	17 (35)
Third-line	21 (36)	17 (35)
>3rd line	14 (23)	12 (26)
EMA restriction of use compliant		
Not compliant	33 (56)	22 (54)
Compliant	26 (44)	26 (46)
Median cycles received, number (range)	5 (1–6)	4 (1–6)
Completion of 3 cycles		
Yes	46 (78)	35 (73)
No	13 (22)	13 (27)
Completion of 6 cycles		
Yes	23 (39)	18 (38)
No	36 (61)	30 (62)
Prior chemotherapy		
Yes	35 (59)	28 (58)
Docetaxel	19 (32)	15 (31)
Docetaxel and Cabazitaxel	16 (27)	13 (27)
No	24 (41)	20 (42)

ECOG: Eastern Cooperative Oncology Group, PSA: prostate-specific antigen, ALP: alkaline phosphatase, Ra-223: Radium 223, EMA: European Medicines Agency.

**Table 2:** Systemic inflammation indexes, FDG-derived parameters, and clinical characteristics in the prediction of OS.

Biomarkers	Univariate Analyses on Complete Cases (*n* = 48)			Multivariate Analyses on Complete Cases (*n* = 48)		Multivariate Analyses on All Aample (*n* = 59)	
	HR (95% CI)	*p* Value	*c*-Index	HR (95% CI)	*p* Value	HR (95% CI)	*p* Value
**Inflammatory biomarkers**							
NLR (1-unit)	1.08 (1.01–1.17)	0.042	0.63	1.09 (1.00–1.20)	0.049	1.09 (1.01–1.19)	0.025
d-NLR (1-unit)	1.27 (1.02–1.58)	0.036	0.65				
LMR (1-unit)	0.95 (0.74–1.21)	0.67	0.57				
PLR (100-unit)	1.03 (0.90–1.18)	0.63	0.56				
SII (100-unit)	1.02 (0.99–1.05)	0.25	0.58				
**FDG-PET parameters**							
SUV max (1-unit)	1.04 (0.96–1.14)	0.33	0.53				
MTV (1-unit on log scale)	2.23 (1.52–3.25)	<0.001	0.75	1.60 (1.09–2.34)	0.016	1.74 (1.22–2.50)	0.002
TLG (1-unit on log-scale)	2.06 (1.46–2.90)	<0.001	0.75				
**Patients’ characteristics**							
ECOG PS							
0–1	1.00 (ref)	<0.001	-	1.00 (ref)	<0.001	1.00 (ref)	<0.001
2	13.4 (5.29–33.74)			7.92 (2.74–22.90)		7.37 (2.94–18.47)	
Gleason group							
<3	1.00 (ref)	0.26	-				
≥3	1.63 (0.70–3.82)						
Lymph node metastases							
No	1.00 (ref)	0.007	-	1.00 (ref)	0.039	1.00 (ref)	0.002
Yes	2.89 (1.33–6.29)			2.66 (1.05–6.75)		3.76 (1.65–8.56)	
*N°* bone metastases							
<6	1.00 (ref)		-				
6–20	1.63 (0.46–5.76)	0.45					
>20	2.66 (0.74–9.53)	0.13					
ALP							
<220	1.00 (ref)	0.001	-				
≥220	3.79 (1.70–8.49)						
**Treatment characteristics**							
Radium therapy line							
1–2	1.00 (ref)	0.92	-				
≥3	1.04 (0.50–2.14)						
Previous chemotherapy							
No	1.00 (ref)	0.44	-				
Yes	1.33 (0.64–2.76)						

NLR: neutrophil to lymphocyte ratio, d–NLR: derived NLR, LMR: lymphocyte to monocyte ratio, PLR: platelet to lymphocyte ratio, SII: systemic inflammation index, FDG–PET: fluorodeoxyglucose positron emission tomography, SUV: standardized uptake value, MTV: metabolic tumor volume, TLG: total lesion glycolysis, ECOG: Eastern Cooperative Oncology Group, PS: performance status, N°: number, ALP: alkaline phosphatase, Ra–233: radium–223.

## Supplementary Materials

The following are available online at https://www.mdpi.com/2072-6694/12/11/3213/s1, Figure S1: Bland-Altman plots comparing MTV (Panel A) and TLG (Panel B) measured by two different groups of PET readers.
