# Supplementary material for: The Prognostic Role of Baseline Metabolic Tumor Burden and Systemic Inflammation Biomarkers in Metastatic Castration-Resistant Prostate Cancer Patients Treated with Radium-223: A Proof of Concept Study
Source: Cancers (Basel). 2020 Oct 31;12(11):3213. doi: 10.3390/cancers12113213 (PMC7693606; doi:10.3390/cancers12113213)
Supplement: Supplementary file 1 [file cancers-12-03213-s001.pdf]

Supplementary Materials:

The Prognostic Role of Baseline Metabolic Tumor Burden and Systemic Inflammation Biomarkers in Metastatic Castration-Resistant Prostate Cancer Patients Treated with Radium-223: A Proof of Concept Study

Matteo Bauckneht, Sara Elena Rebuzzi, Alessio Signori, Maria Isabella Donegani, Veronica Murianni, Alberto Miceli, Roberto Borea, Stefano Raffa, Alessandra Damassi, Marta Ponzano, Fabio Catalano, Valentino Martelli, Cecilia Marini, Francesco Boccardo, Silvia Morbelli, Gianmario Sambuceti and Giuseppe Fornarini

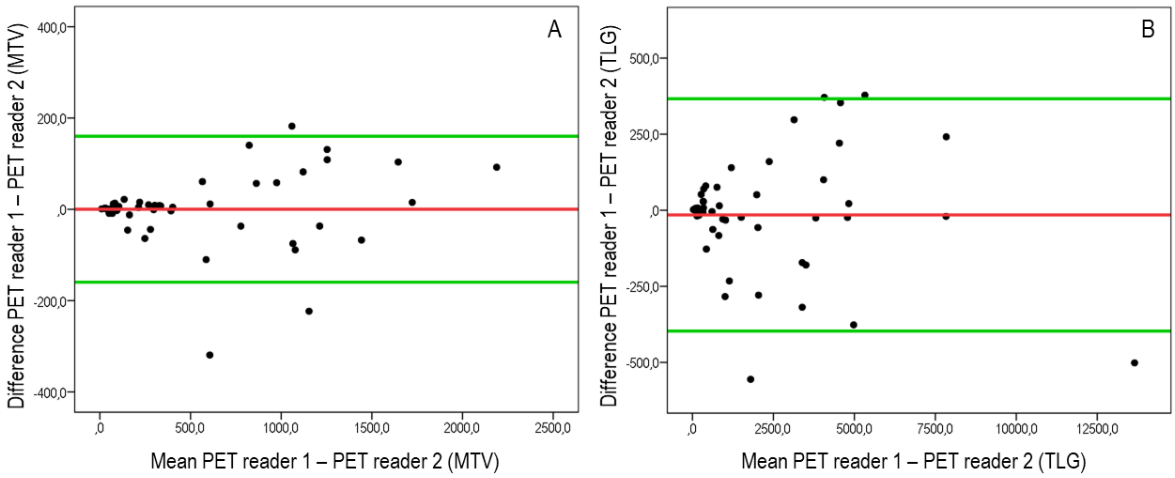

Figure S1. Bland-Altman plots comparing MTV (Panel **A**) and TLG (Panel **B**) measured by two different groups pf PET readers. The horizontal axis represents the mean of the two measurements and the vertical axis the difference between them. The ref line shows the median difference (close to zero) and the green lines show the 95% limits of agreements. In both cases, the median is very close to zero, indicating no systematic bias, and the range of limits of agreements is relatively small compared to the scale of the MTV or TLG values, indicating a good agreement among the majority of patients.
